# The Action of Quinine upon the Tertian, Quartian and Estivo-Autumnal Malarial Plasmodia

**Published:** 1907-01

**Authors:** 


### THE ACTION OF QUININE UPON THE TERTIAN, QUARTIAN, AND ESTIVO-AUTUMNAL MALARIAL PLASMODIA.

Craig, who has done such excellent work upon the subject of malarial infection, has contributed to *American Medicine* for May a paper on this subpect. A great deal has been written regarding the administration of quinine in the treatment of malarial infection, some authorities maintaining that it should always be given in divided doses, while others claim better results from the administration of one large dose before the expected paroxysm. It is not necessary to discuss at present the many contributions dealing with this subject, but those interested should consult the very valuable paper of Dock, which contains a summary of the opinions of the various writers upon the administration of quinine in malaria.

As shown, however, in this study, quinine acts injuriously upon the plasmodia in all stages of their human-life cycle, with the possible exception of the large, full-grown plasmodium just prior to segmentation, and thus to secure the best results the drug should always be present in the blood. Given in divided doses at regular intervals the blood constantly contains a sufficient quantity practically to stop the development of the plasmodia which have succeeded in escaping it while free in the blood plasma, and recovery is thereby hastened.

The practice of giving quinine in the manner mentioned is not only justified by the morphological changes in the plasmodia herein described, but its value is proved by practical experience. The writer has at present the records of over four hundred cases of malarial fever, observed in soldiers returning from the Philippines and in the Philippines, including tertian, quartian, and estivo-autumnal infections, in which quinine was administered in doses of .50 gramme every three hours until 1.50 to 2.00 grammes were taken, and in all these cases recovery was prompt and occurred more quickly than in similar cases in which one large dose was administered prior to sporulation. In the tertian cases it was but very rarely that a second chill occurred, although in about 20 per cent. of the cases a rise of temperature was noted upon the day of the expected paroxysm. In the quartan cases a second paroxysm never occurred after the beginning of the administration of quinine in this way, while in the estivoautumnal cases, which comprised nearly two-thirds of the total number, the temperature, except in two pernicious cases, reached normal in from two to three days and recovery occurred.

It is in the latter type of malarial infection that the administration of quinine in divided doses is especially efficient, for in this type it is generally impossible to tell the exact time of the ensuing paroxysm, and thus it is impossible to attack the parasites while they are free in the blood plasma by the administration of one large dose of the drug.

From these studies upon the action of quinine upon the plasmodia of malaria the author believes the following conclusions are justified:

1. Quinine exercises an injurious effect upon the plasmodia of malaria during all stages of their human-life cycle, whether intracorpuscular or extracorpuscular, except when it is administered just prior to sporulation, at which time the sporulating body is not injured and sporulation occurs, but most of the spores are desquamated by the drug while they are free in the blood plasma.

2. The marked morphologic changes, degenerative in character, produced by quinine in all species of the malarial plasmodia, during all stages of their growth, prove that in order to secure the best therapeutic results the drug should be continually present in the blood, and this is only possible when it is administered in divided doses at regular intervals of time.—*Therapeutic Gazette.*

